# Methylation pattern of caveolin-1 in prostate cancer as potential cfDNA biomarker

**DOI:** 10.17305/bjbms.2022.7497

**Published:** 2023-01-06

**Authors:** Lucija Škara, Tonći Vodopić, Ivan Pezelj, Irena Abramović, Borna Vrhovec, Alen Vrtarić, Nino Sinčić, Davor Tomas, Stela Bulimbašić, Tomislav Kuliš, Monika Ulamec

**Affiliations:** 1 Department of Biology, University of Zagreb School of Medicine, Zagreb, Croatia; 2 Scientific Group for Research on Epigenetic Biomarkers, University of Zagreb School of Medicine, Zagreb, Croatia; 3 Scientific Centre of Excellence for Reproductive and Regenerative Medicine, University of Zagreb School of Medicine, Zagreb, Croatia; 4 Ljudevit Jurak Clinical Department of Pathology and Cytology, Sestre milosrdnice University Hospital Center, Zagreb, Croatia; 5 Department of Urology, Sestre milosrdnice University Hospital Center, Zagreb, Croatia; 6 Department of Clinical Chemistry, Sestre milosrdnice University Hospital Center, Zagreb, Croatia; 7 Department of Pathology, University of Zagreb School of Medicine, Zagreb, Croatia; 8 Pathology and Cytology Department, University Hospital Center Zagreb, Zagreb, Croatia; 9 Department of Urology, University Hospital Center Zagreb, Zagreb, Croatia

**Keywords:** Prostate cancer (PCa), biomarker, cfDNA methylation, CAV1

## Abstract

High prevalence and mortality of prostate cancer (PCa) are well-known global health issues. Novel biomarkers for better identifying patients with PCa are the subject of extensive research. Prostate-specific antigen (PSA) shows low specificity in screening and diagnostics, leading to unnecessary biopsies and health costs. Eighty patients with PCa and benign prostate hyperplasia (BPH) were included in the study. We analyzed *CAV1* gene expression and methylation in tissue. *CAV1* cfDNA methylation from blood and seminal plasma was accessed as a potential PCa biomarker. Although methylation in blood plasma did not differ between PCa and BPH patients, methylation in seminal plasma showed better PCa biomarker performances than tPSA (AUC 0.63 vs. AUC 0.52). Discrimination of BPH and Gleason grade group 1 PCa patients from patients with higher Gleason grade groups revealed very good performance as well (AUC 0.72). *CAV1* methylation is a useful biomarker with potential for further seminal plasma cfDNA research, but its diagnostic accuracy should be improved, as well as general knowledge about cfDNA in seminal plasma.

## Introduction

Prostate cancer (PCa) is the most common malignancy in men worldwide [1]. The suspicion of PCa arises from abnormal digitorectal examination and/or elevated prostate-specific antigen (PSA) which are indications for needle biopsy with a final pathohistological diagnosis [2]. PSA is not tumor-specific marker since it can be elevated in other conditions, such as benign prostatic hyperplasia (BPH), prostatitis, and other non-malignant conditions. With widespread PSA screening there is a concern of overdiagnosing and overtreatment of clinically indolent PCa. There is also a small subset of PCa patients without PSA elevation. It is also worth mentioning that clinically and biologically, there are two different faces of the same disease—indolent and aggressive. Indolent form concerning PCa with very slow progression mostly regarding Gleason group 1 and/or 2. There are some theories that Gleason group 1 PCa should be reclassified as premalignant instead of the invasive lesion [3]. The aggressive form is characterized by early metastatic events and demanding therapeutic approaches. Due to low PSA specificity, many asymptomatic PCas with low clinical risk factors for progression are detected and treated while, at the same time, clinically significant cases may remain undiagnosed [2, 4].

There is a need for more specific PCa biomarker. Since assessing the tissue methylation of three genes with ConfirmMDx test improves the detection of false-negative biopsy, our research is focused on methylation patterns in liquid biopsy samples while DNA originating from a tumor cell and tumor microenvironment can be found in liquid outside the cell [5]. We assessed the methylation pattern of Caveolin-1 (*CAV1*) since a previous study showed higher methylation of PCa tissue than adjacent normal prostate tissue [6]. *CAV1* protein expression significantly differs between PCa and BPH and does not correlate with preoperative PSA levels, but its overexpression has been associated with advanced clinical stage, metastasis, angiogenesis, androgen insensitivity, increased risk of aggressive PCa recurrence after surgery, and poor survival [7–10]. CAV1 plays an important role in signaling.

CAV1 is a protein crucial for caveolae formation in the plasma membrane. Through its caveolin scaffolding domain, CAV1 interacts with numerous signaling molecules. It is involved in the regulation of proliferation, apoptosis, differentiation, migration, angiogenesis, ion channel activity, endocytosis, senescence, and mechanosensing [11–15]. *CAV1* expression differs among various cancer types and exhibits the dual role of this protein [13, 16]. It behaves as a tumor promoter and tumor suppressor. It is possible that during cell transformation *CAV1* expression drops and then increases in tumor progression, metastasis, and drug resistance [13].

Based on these facts, we estimated that *CAV1* could improve PCa diagnostics, thus, the aim of this study is to explore *CAV1* methylation of cfDNA in liquid biopsy samples, blood, and semen, as a potential minimally invasive marker or as part of a diagnostic panel to distinguish PCa from BPH or indolent from clinically significant PCa. We also examined methylation and protein expression in the tissue to compare and supplement results from liquid biopsy samples.

## Materials and methods

### Patients and sample collection

Eighty patients scheduled for transrectal prostate biopsy due to clinical suspicion of PCa were included in the study. Biopsy was performed from October 2018 to October 2021 at the Urology Department of the University Hospital Center Sestre Milosrdnice and University Hospital Center Zagreb. Prior biopsy, peripheral venous blood samples and semen were obtained. Blood was collected in 6 ml EDTA-treated tubes (Vacuette^®^ , Greiner Bio-One GmbH, Kremsmünster, Austria) and centrifuged at 1400× *g* for 10 min at room temperature. To avoid contamination with cellular DNA, supernatants were carefully collected and centrifuged at 4500×*g* for 10 min at room temperature.

Semen samples were collected in the urine cup by masturbation 3–5 days after sexual abstinence. Semen was centrifuged at 400× *g* for 10 min at room temperature. To avoid contamination with cellular DNA, supernatants were carefully collected and centrifuged at 12,000× *g* for 10 min at room temperature. All blood and semen fractions were stored at −80 ^∘^C until analysis was performed. Following biopsy, PCa or BPH was diagnosed by an institutional uropathologist. Tumors were graded and staged according to the Gleason Score (GS) and WHO 2016/ISUP (International Society of Urological Pathology) grade groups. Blood and seminal fractions were stored at −80 ^∘^C until analysis.

The study was approved by the Ethical Committees of the University Hospital Center Sestre Milosrdnice, University Hospital Center Zagreb, and University of Zagreb School of Medicine. The research was conducted in accordance with the Declaration of Helsinki. All patients were informed about the details of the study, and written informed consent was obtained from each patient before enrolment into the study.

**Figure 1.**
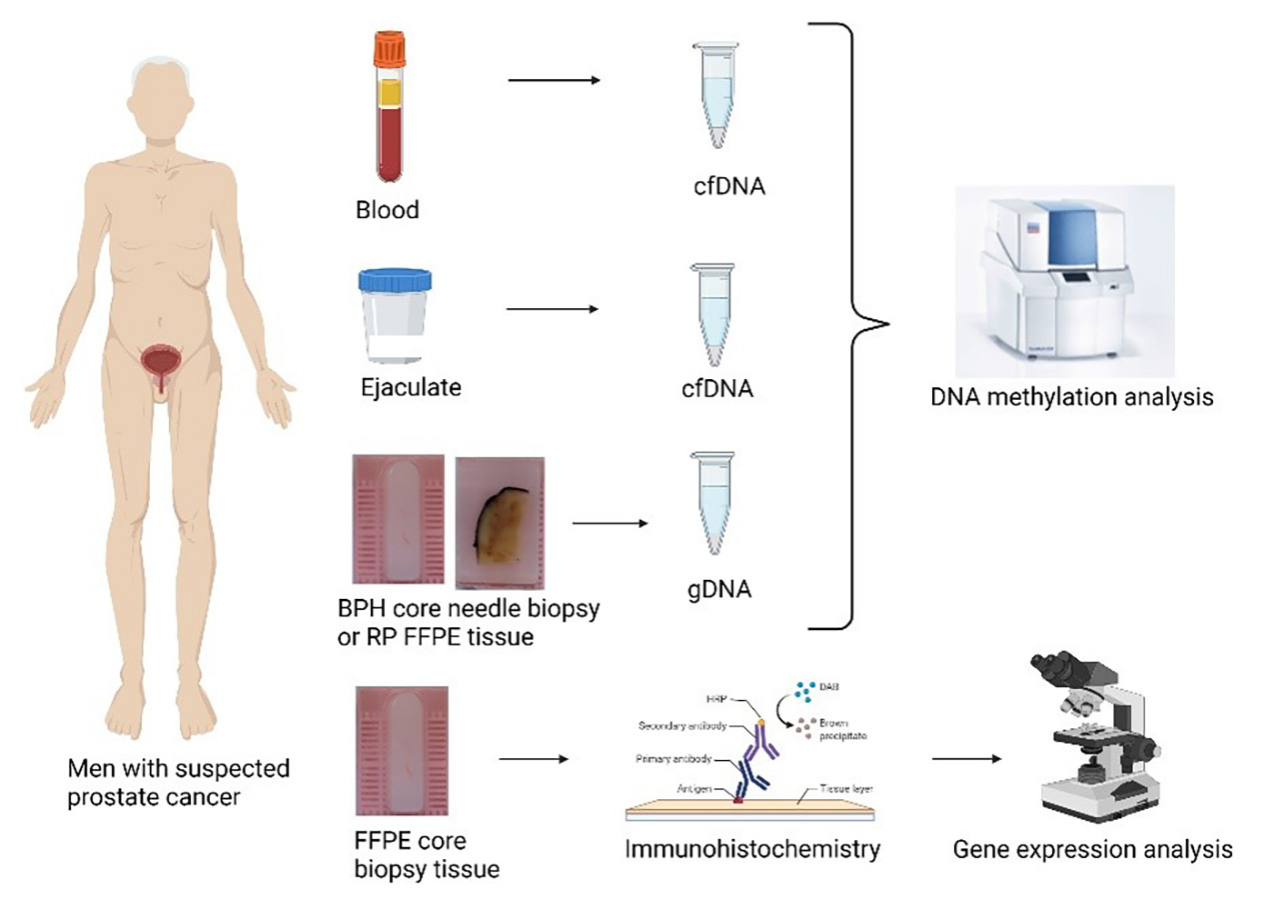
**Overview of the experimental workflow.** BPH: Benign prostatic hyperplasia; RP: Radical prostatectomy. Created with BioRender.

In total, 80 patients were included in this study, and according to histopathological analysis, patients were divided into two groups: 40 in PCa and 40 in the BPH group. From BPH patients, two core biopsy formalin-fixed paraffin-embedded (FFPE) blocks containing glands and stroma were selected for further analysis (one for hematoxylin and eosin (HE) staining and immunohistochemistry (IHC) and another one for gDNA isolation). From PCa patient, one core biopsy was selected for HE staining and IHC. Due to small size of core biopsy, it was impossible to precisely separate surrounding non-tumorous tissue (NTT) from PCa tissue. Thus from patients enrolled in the study who underwent radical prostatectomy in collaborative hospital centers, one representative paraffin block of radical prostatectomy containing at least 30% of tumorous tissue and NTT was selected for HE staining and gDNA isolation (*N* ═ 29). NTT refers to morphologically normal glands and stroma, with exception of intraductal cancer or prostatic intraepithelial neoplasia. Tissue fractions consisted of epithelial and stromal cells together, without separation on cell types. Experimental workflow is presented in Figure 1. Unfortunately, we did not obtain paraffin block of radical prostatectomy tissue from patients who underwent radical prostatectomy outside our collaborative hospital centers and thus we miss their gDNA methylation data.

Besides sample material, clinical (PSA level in serum, Gleason Score (GS), WHO 2016/ISUP grade group) and epidemiological data [family history of PCa and cancer in general, age, smoking habits, body mass index (BMI)] were obtained.

### FFPE tissue processing and IHC

For HE staining and IHC, one core biopsy FFPE block from each patient was sectioned at 4 µm. HE stained slides were examined from two uropathologists (U.M., T.D.) to confirm the diagnosis and to identify lesions and cells (PCa glands, NTT, BPH glands, epithelium, stroma). They also marked radical prostatectomy tissue slides for micro-dissection (PCa and NTT). One core biopsy slide from each patient was further used for IHC staining. FFPE slides were deparaffinized in xylene and rehydrated in a series of decreasing alcohol solutions. Heat-induced epitope retrieval was performed using a vegetable steamer (Tris/EDTA pH 9.0, 20 min). Slides were blocked with 5% goat serum following overnight incubation at 4 ^∘^C with polyclonal primary antibody against CAV1 (HPA049326, 1:2 500) diluted in 1% BSA/TBS/0.1% Tween-20. Slides were incubated in the dark with 3% H_2_O_2_ for 20 min to block endogenous peroxidase activity. The next step involved the application of secondary antibody (Dako REAL EnVi-sion Detection System, K5007, Agilent Technologies) and incubation at 37 ^∘^C for 1 h, followed by a serial TBS wash three times for 5 min. Subsequently, the samples were incubated for 6 min in DAB (3,3-diaminobenzidine-tetrahydrochloride) (Dako REAL EnVision Detection System, K5007, Agilent Technologies). Slides were counterstained with hematoxylin. Positive (kidney), negative (spleen), and no primary antibody control were used as quality controls. Slides were analyzed under microscope Olympus BX51. Staining intensity and staining proportion were semi-quantified in epithelium and stroma separately, by two uropathologists (U.M., T.D.), according to Table 1. The staining score was calculated by multiplying the staining proportion score and the staining intensity score and all disagreements were resolved by join committee.

**Table 1:** Semi-quantified scoring system

**Percentage of positive cells**	**Staining proportion score**	**Staining intensity**	**Staining intensity score**
0%, no stained cells	0	no signal	0
1%–25% of cells stained	1	weak	1
>25%–50% of cells stained	2	moderate	2
>50%–75% of cells stained	3	strong	3
>75% of cells stained	4		

### DNA extraction and bisulfite conversion

For gDNA isolation and methylation analysis, one FFPE block from BPH patient was sectioned at 10 µm. FFPE block containing radical prostatectomy specimen was sectioned at 4 µm for HE staining and marked for PCa and NTT area and then cut at 10 µm for gDNA isolation and methylation analysis. gDNA was extracted from 10 µm slices of FFPE tissue following protocol described by Talukdar et al. [17]. gDNA was isolated from prostate cores with BPH tissue without separation, while radical prostatectomy FFPE tissue was manually microdissected. Tumor tissue was separated from surrounding NTT using surgical blades and HE-stained slide as a guide. gDNA was isolated from the tumor and NTT. Isolated gDNA was quantified by spectrophotometry (NanoDrop ND-2000, NanoDrop Technologies, USA) and stored at −80 ^∘^C.

After thawing, seminal plasma was centrifuged at 20,000 *g* for 10 min. Blood plasma cfDNA was isolated with the NucleoSnap cfDNA kit (from blood plasma and seminal plasma > 1 ml) and Nucleospin (seminal plasma < 1 ml) (MACHERY-NAGEL, Germany) using QIAvac 24 Plus vacuum station (QIAGEN, Germany) as previously published [19]. Isolated cfDNA was quantified by qPCR on the CFX96 Touch Real-Time PCR Detection System (Bio-Rad Laboratories) using SsoAdvanced Universal SYBR Green Supermix (Bio-Rad Laboratories) and primers for 82–bp LINE-1 fragment as previously published [18].

The bisulfite conversion of cfDNA and gDNA is carried out with EpiTect Plus DNA Bisulfite Kit (QIAGEN, Germany) according to the manufacturer’s instructions.

### PCR and pyrosequencing

Bisulfite-treated DNA was amplified using PyroMark PCR kit (QIAGEN, Germany) (Table 2). PCR and sequence primers for pyrosequencing were designed with PyroMark Assay Design 2.0 (QIAGEN, Germany). Primer sequences are listed in Table 3. *CAV1* methylation was analyzed by pyrosequencing using Pyromark Q24 Advanced System with PyroMark Q24 CpG Advanced Reagents (QIAGEN, Germany) according to the manufacturer’s instructions. Briefly, in a skirted 24-well PCR plate 1 µl streptavidin beads (GE Healthcare, UK), 39 µl PyroMark binding buffer (QIAGEN, Germany), 20 µl high-purity water, and 20 µl PCR product were mixed and plate shook for a minimum of 10 min at 1400 rpm. By using the vacuum workstation, amplicons were denatured and transferred into a pyrosequencing plate containing sequencing primer. After heating the pyrosequencing plate for 3 min at 80 ^∘^C, the plate was inserted into the pyrosequencer. Results were analyzed by the PyroMark Q24 Advanced Software 3.0.1. Analyzed sequence encompasses nine CpG sites (hg38; chr7:116,524,607-116,524,746) along EH38E2583972 ENCODE Candidate Cis-Regulatory Element (promoter). Average methylation of sample was calculated from nine CpG and also used in comparison. Which CpG from our study corresponds to which Illumina cg designations is listed in supplementary (Table S1).

**Table 2:** PCR cycling conditions

**Steps**	**T (^∘^C)**	**Time**	**No. of cycles**
Activation	95 ^∘^C	15 min	1
Denaturation	94 ^∘^C	30 sec	
Annealing	52.5 ^∘^C	30 sec	45
Extension	72 ^∘^C	30 sec	
Final extension	72 ^∘^C	10 min	1

**Table 3:** Primer sequences used in this study

Forward primer	GTTTAGGATAGGGTAGGATTGTG
Reverse primer	Biotin - ACCTAAAACAACATTTTCCCTACT
Sequencing primer	GGGTAGGATTGTGGAT
Sequence to analyze	TGTTTTTGTY GTTTTGGTTG TTTATATTGG GTATTTTTGT AGGYGYGTYG GTTTTTTTTA TTTTTGTTGA GATGATGTAT TGYGAAAATA TTYGTTTTTT TYGGGAYGTT TTTYGGTGGT TTAGAGTAGG GAAAATGTTG

### Ethical statement

The study was conducted according to the guidelines of the Declaration of Helsinki, and approved by the Ethics Committee of University of Zagreb School of Medicine (protocol code 641-01/18-02/01, 25 January 2018), University Clinical Hospital Center Sestre milosrdnice (protocol code EP-18327/17-2, 7 December 2017) and University Clinical Hospital Center Zagreb (protocol code 8.1-17/213-2, 11 December 2017).

**Table 4:** Baseline characteristics of patients included in the study

		**PCa**	**BPH**	***P* value**
Number		40	40	
Age (years)	median (range)	61 (44–73)	60 (46–72)	0.820
	≤49	4	3	
	50–59	15	15	
	60–64	11	14	
	65–69	8	4	
	≥70	2	4	
tPSA (ng/ml)	Median (range)	6.660 (1.81–63.16)	7.360 (2.4–21.57)	0.795
Gleason grade group	1 (Gleason score 3+3)	7		
	2 (Gleason score 3+4)	26		
	3 (Gleason score 4+3)	6		
	5 (Gleason score 4+5)	1		
Smoking habits	never smoker	18	17	0.947
	ex-smoker	17	12	
	current smoker	3	5	
	unknown	2	6	
BMI (kg/m^2^)	median (range)	27.7 (24.2–38.4)	27.5 (20.8–34.9)	0.612
	unknown	2	6	

BPH: Benign prostatic hyperplasia; PCa: Prostate cancer; BMI: Body mass index; tPSA: Total prostate-specific antigen.

### Statistical analysis

Statistical analysis was performed using GraphPad Prism (GraphPad Software, Inc., San Diego, CA, USA). Patient characteristics are presented using descriptive statistics. To compare *CAV1* protein expression and methylation between PCa and NTT tissue Wilcoxon matched-pairs signed-rank test was used, while for PCa-BPH and NTT-BPH comparison Mann–Whitney test was used. Kruskal–Wallis test with *post-hoc* test Dunn’s multiple comparison test was used to compare cfDNA methylation across different pathological states, year groups, smoking status. Mann–Whitney test was used to compare baseline characteristics, *CAV1* methylation in blood and semen of PCa and BPH patients. Spearman rank correlation was used to measure the strength of association between expression, methylation, clinical, and epidemiological characteristics. The sensitivity and specificity of the *CAV1* methylation were assessed with a receiver operating characteristic (ROC) curve. *P* values < 0.05 were considered significant. Fisher’s exact test was used to assess the association between positive family history and diagnosis (https://www.graphpad.com/quickcalcs/contingency1/).

## Results

### Clinical and pathological data

Forty patients with a histopathologically confirmed diagnosis of PCa and 40 patients with a histopathologically confirmed diagnosis of BPH were included. PCa and BPH group were similar in age, tPSA value, smoking habits, and BMI. In the PCa group, the median age was 61 (44–73) while in the BPH group 60 (46-72). Most PCa and BPH patients were in age group 2 (50–59). The median PSA value of the PCa group was 6.66 ng/ml (range 1.81–63.16), while of the BPH group 7.685 ng/ml, (range 2.4–21.57). PCa patients were histologically classified into four Gleason grade groups (GGG): 1 (7 cases), 2 (26 cases), 3 (6 cases), and 5 (1 case). Baseline characteristics are presented in Table 4.

### *CAV1* expression in tissue

**Figure 2.**
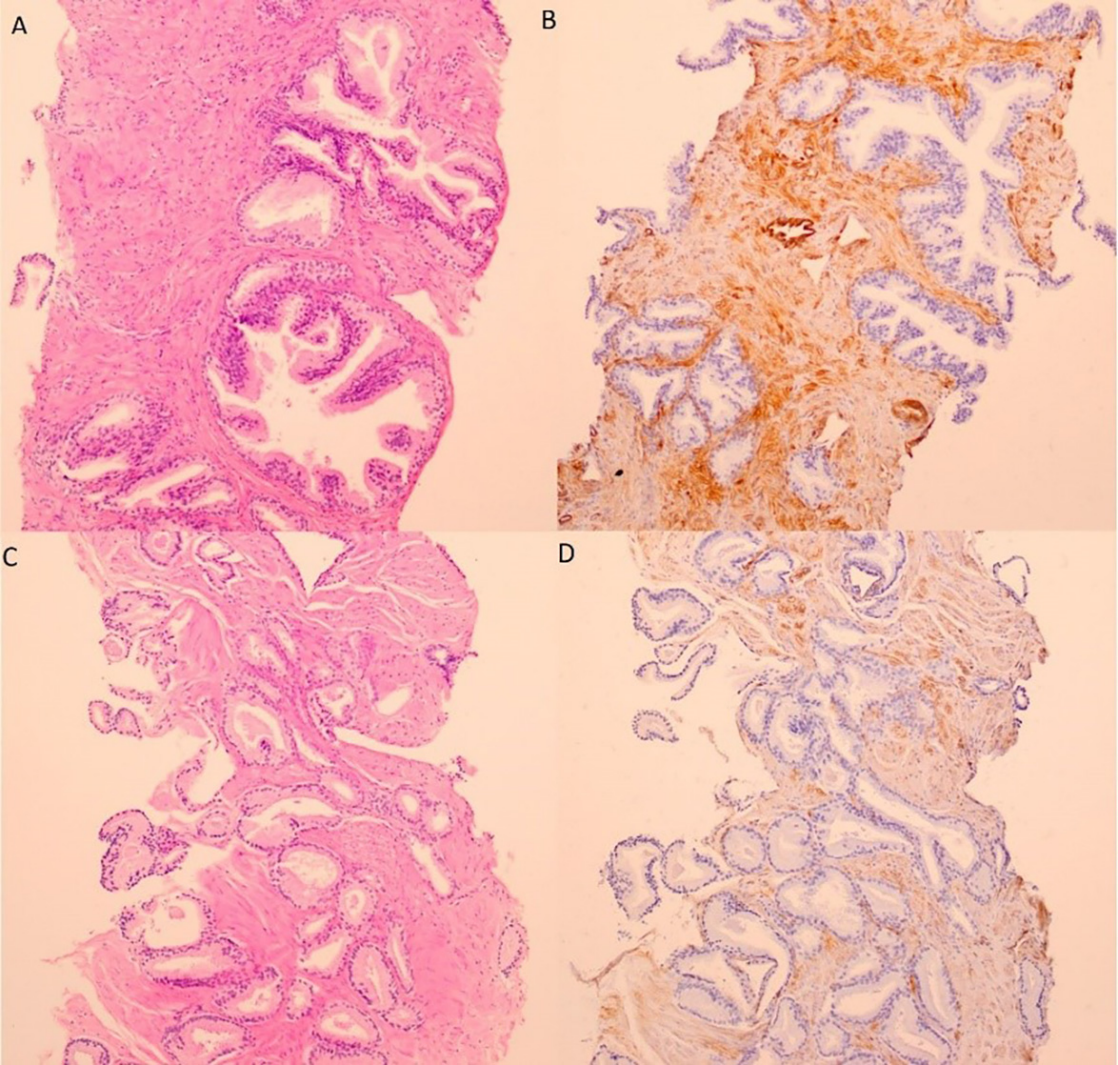
Hematoxylin-eosin staining (A, C) and immunohistochemical staining for CAV1 (B, D) in benign prostatic hyperplasia (A, B) and prostate cancer (C, D) biopsy cores. 100x.

Except one NTT tissue (staining score ═ 1), prostate epithelial cells did not express *CAV1* regardless of histopathological diagnosis (Figure 2). The intensity, staining proportion, and staining scores were higher in BPH stoma (medians: 3, 3, 6) than in PCa stroma (medians: 2, 2, 4). Expression in NTT stroma tissue was similar to BPH (medians: 3, 2, 6). None of the samples had >75% of stromal cells stained (staining proportion score 4). In more than a half BPH stroma samples (21/40) staining proportion score was 3, compared to PCa stroma samples (10/39). Staining score ≥ 4 was find in 20/39 PCa stroma samples, 26/39 NTT stroma samples, and 31/40 BPH samples.

**Figure 3.**
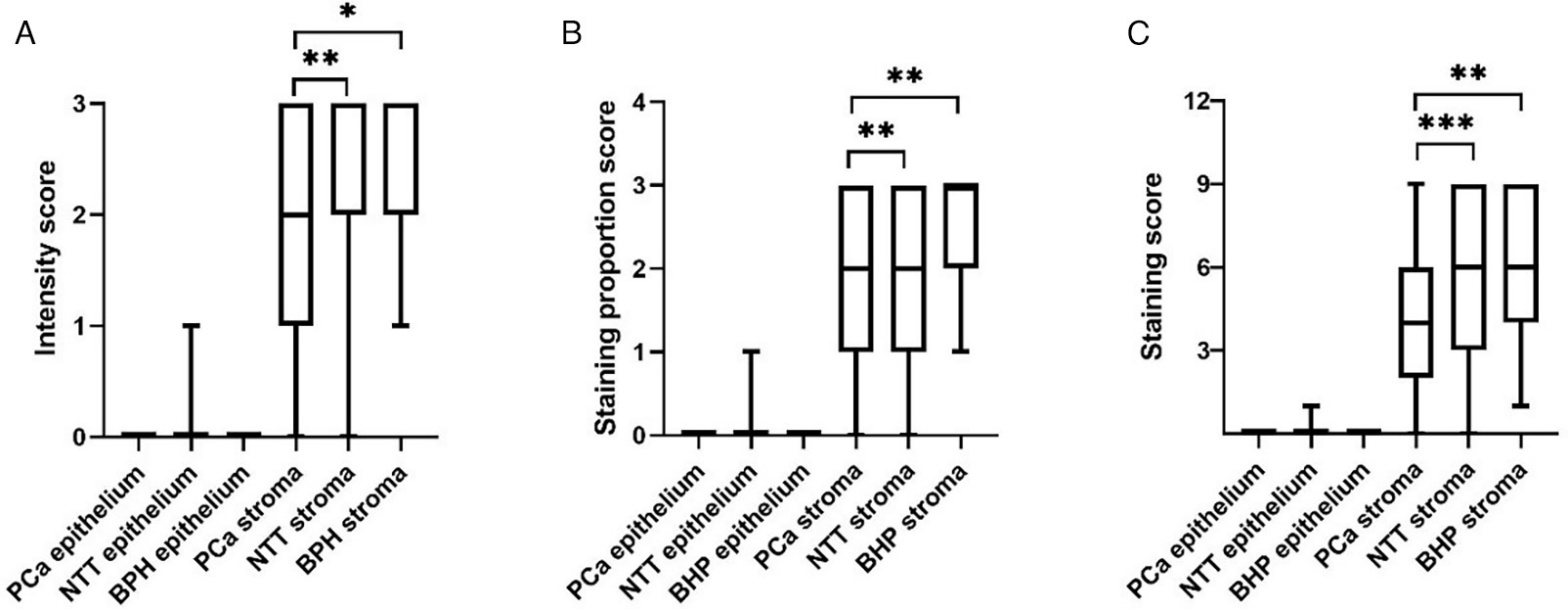
Boxplots graphs showing the staining intensity score (A), staining proportion score (B), and staining score (C) of *CAV1* expression across PCa, NTT, and BPH tissue (median, interquartile range, minimum, maximum). Wilcoxon matched-pairs signed-rank test, Mann–Whitney test. *P ≤ 0.05, **P ≤ 0.01, ***P ≤ 0.001. BPH: Benign prostatic hyperplasia; PCa: Prostate cancer; NTT: non-tumorous tissue.

BPH stroma showed a statistically significant higher intensity score (*P* ═ 0.046), staining proportion score (*P* ═ 0.008), and staining score (*P* ═ 0.002) than PCa stroma (Figure 3). Wilcoxon matched-pairs signed-rank test showed that staining intensity score (*P* ═ 0.002), staining proportion score (*P* ═ 0.009), and staining score (*P* ═ 0.001) were statistically significant different between PCa and their matched pair NTT. There was no significant difference between NTT and BPH. Staining score did not correlate with Gleason grade group (*r* ═ 0.13, *P* ═ 0.450) nor with tPSA (*r* ═ 0.03, *P* ═ 0.783). We also noticed higher staining intensity in the stroma from the central prostate. *CAV1* was also highly expressed in atrophic prostate glands and blood vessel endothelial cells.

### Methylation of *CAV1* in tissue

**Figure 4.**
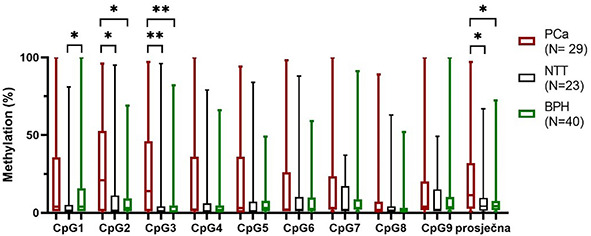
**Box plot presenting methylation percentage of each CpG site and average methylation in PCa, NTT, and BPH tissue.** Box plot indicates median, interquartile range, minimum and maximum. Wilcoxon matched-pairs signed-rank test, Mann–Whitney test. PCa: Prostate cancer; NTT: Non-tumor tissue; BPH: Benign prostatic hyperplasia. *P ≤ 0.05, **P ≤ 0.01.

PCa had the highest gDNA methylation mean of all CpG sites and average methylation. PCa had the highest median of 4 CpGs and average, while the median methylation of 4 CpGs was the same as BPH. Only at CpG6, BPH median methylation was slightly higher than PCa median (Figure 4). Overall, methylation among analyzed tissue types did not differ significantly in six CpGs.

**Figure 5.**
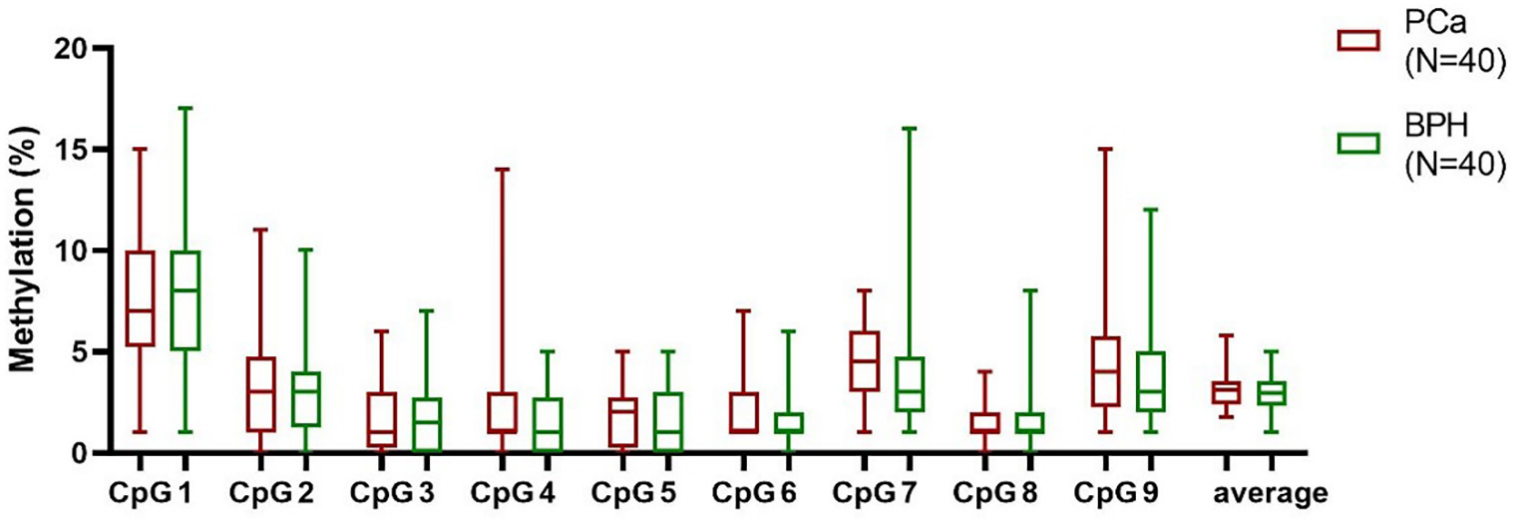
**Box plot presenting methylation percentage of each CpG site and average methylation in PCa, NTT, and BPH tissue.** Box plot indicates median, interquartile range, minimum and maximum. Wilcoxon matched-pairs signed-rank test, Mann–Whitney test. PCa: Prostate cancer; NTT: Non-tumor tissue; BPH: Benign prostatic hyperplasia. *P ≤ 0.05, **P ≤ 0.01.

**Figure 6.**
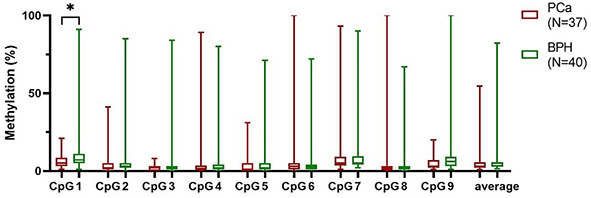
**Box plot presenting methylation percentage of each CpG site and average methylation in cfDNA from seminal plasma of patients with PCa and BPH.** Box plot indicates median, interquartile range, minimum and maximum. Mann–Whitney U test. PCa: Prostate cancer; BPH: Benign prostatic hyperplasia. *P ≤ 0.05.

**Figure 7.**
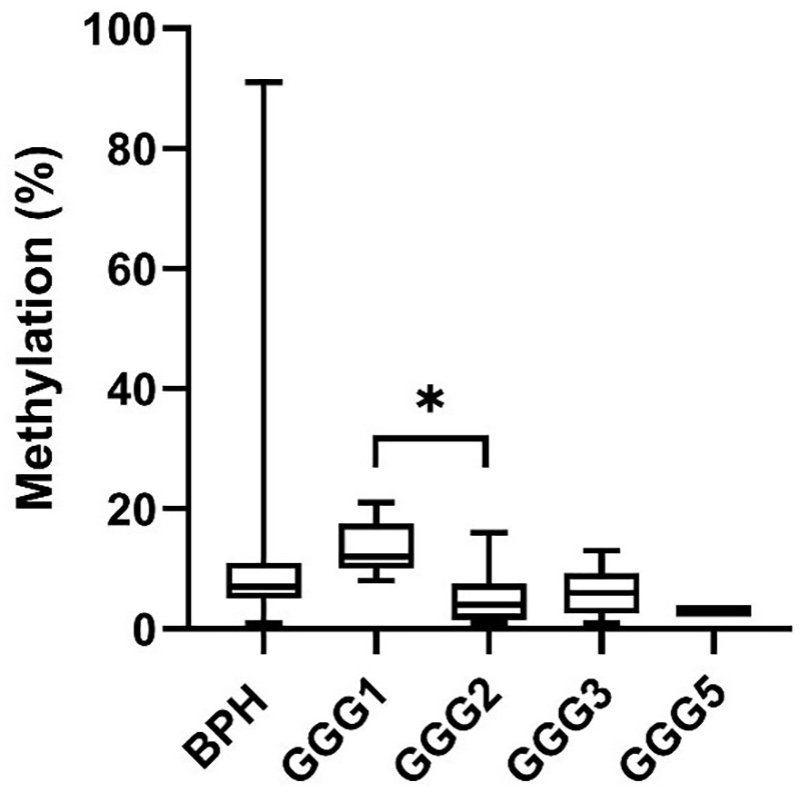
**Box plot presenting methylation percentage of cfDNA from seminal plasma at CpG1 site in patients with PCa and BPH**. Box plot indicates median, interquartile range, minimum and maximum. Kruskal–Wallis test with post-hoc test Dunn's multiple comparison test. BPH: Benign prostatic hyperplasia; GGG: Gleason grade group; PCa: Prostate cancer. *P ≤ 0.05.

The statistically significant difference between tissue types was found in the first three CpG sites and in average methylation. The methylation median value of CpG1 in PCa and BPH tissue was 4%, while in NTT 1%. The difference between NTT and BPH was statistically significant (*P* ═ 0.030). The methylation median of CpG2 and CpG3 was the highest in PCa (21% and 14%). Methylation of CpG2 and CpG3 was significantly higher in PCa then BPH (*P* ═ 0.011, *P* ═ 0.009) and NTT (*P* ═ 0.015, *P* ═ 0.002). Also, average methylation was significantly higher in PCa than in NTT (*P* ═ 0.039) and BPH (*P* ═ 0.027). We did not find any correlation between staining score and tissue methylation.

### cfDNA methylation of *CAV1* in blood plasma

The difference in all cfDNA methylation mean and median between groups was ≤2% (Figure 5). There was no statistically significant difference in blood cfDNA methylation between PCa and BPH patients. Also, there was no statistically significant difference when considering blood CpGs methylation regarding benign or malignant lesions with low toward high grade lesions. There was no correlation between benign or malignant lesions or GGG and blood cfDNA methylation.

#### cfDNA methylation of *CAV1* in seminal plasma

Seminal cfDNA methylation of 8 CpGs and average methylation was similar, and difference between groups was ≤1% (Figure 6). Statistically significant difference between PCa and BPH group was found only in CpG1. Methylation median values of CpG1 in PCa and BPH tissue were 5% and 7%, and difference was statistically significant (*P* ═ 0.043).

When considering seminal CpG1 methylation regarding benign or malignant lesions with low toward high grade lesions, methylation of GGG1 samples was statistically significantly more methylated than GGG2 samples (*P* ═ 0.012) (Figure 7). There was no significant difference in methylation of other CpGs nor of average methylation regarding pathological diagnosis.

An analysis of the correlation between benign or malignant lesions with low toward high grade lesions and seminal cfDNA methylation demonstrated a weak negative correlation at CpG1 (*r* ═ −0.29, *P* ═ 0.009) and CpG9 (*r* ═ −0.24, *P* ═ 0.041) while there was no correlation between benign or malignant lesions with low toward high grade lesions and other CpGs or average methylation. When comparing GGGs with seminal cfDNA methylation, moderate negative correlation was present at CpG7 (*r* ═ −0.37, *P* ═ 0.025).

### Correlation of methylation status in the tissue with blood and seminal plasma

Spearman correlation analysis was used to evaluate the correlation between tissue gDNA methylation and liquid biopsy cfDNA methylation. Since methylation of PCa and NTT tissue was analyzed separately, and correlation analysis was performed for both data sets. Results are presented in Table 5. Positive correlations were observed between tissue and seminal methylation at positions CpG7 and CpG8. Moreover, NTT gDNA methylation showed stronger correlation with seminal cfDNA than PCa gDNA. There was no correlation observed at other CpG positions or between gDNA and cfDNA from blood plasma.

### Biomarker performances of seminal *CAV1* CpG1 methylation and tPSA

**Figure 8.**
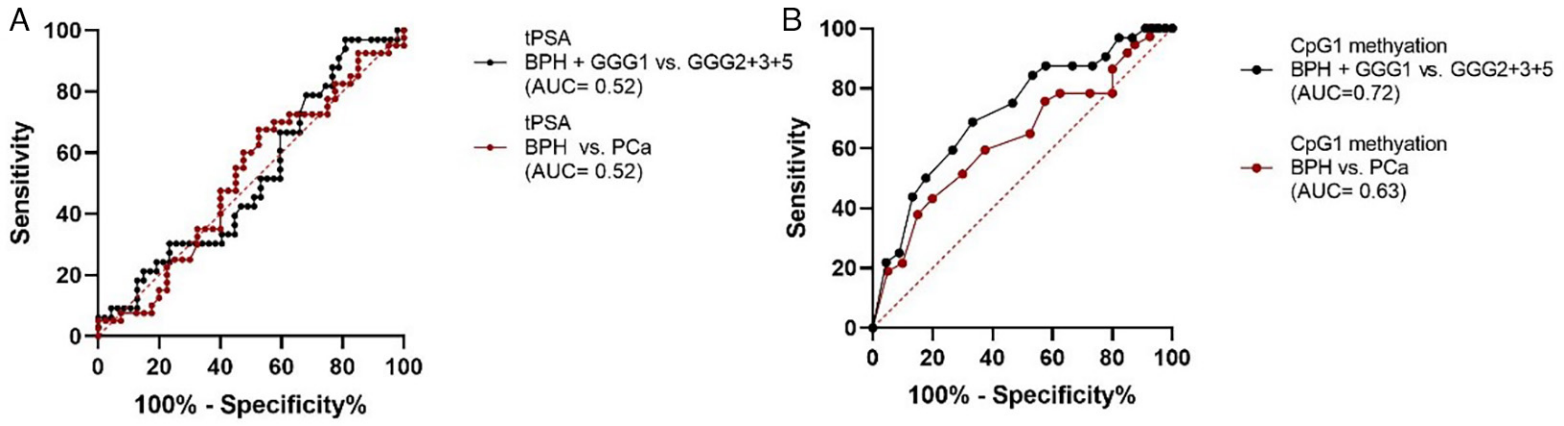
ROC curve analyses of tPSA (A) and seminal *CAV1* CpG1 methylation (B). ROC curve analyses were performed by comparing BPH patients with PCa patients and by comparing regrouped BPH and GGG1 patients with regrouped GGG2, GG3, and GGG5 patient. BPH: Benign prostatic hyperplasia; PCa: Prostate cancer; NTT: Non-tumorous tissue; GGG: Gleason grade group.

ROC curve analyses were performed using data obtained from patients involved in this study (CpG1 mathylation in seminal plasma and tPSA values). Diagnostic sensitivity (SEN), specificity (SPE), and area under the curve (AUC) of tPSA and CAV1 CpG1 methylation of cfDNA in seminal plasma were compared. When comparing BPH with PCa, AUC value for tPSA (AUC 0.52, 95% CI: 0.39–0.65, *P* ═ 0.777, SEN 68%, SPE 48%) was lower than AUC for CpG1 methylation (AUC 0.63, 95% CI: 0.51–0.75, *P* ═ 0.044, SEN 59%, SPE 63%) (Figure 8). If grouping BPH and GGG1 together and comparing it with GGG ≥2, AUC value for CpG1 methylation (AUC 0.72, 95% CI: 0.61–0.84, *P* ═ 0.001, SEN 69%, SPE 67%) was higher than tPSA (AUC 0.52, 95% CI: 0.39–0.65, *P* ═ 0.762, SEN 97%, SPE 19%).

### Lifestyle and cancer family history

Age did not correlate with methylation in seminal plasma. When patients were divided by diagnosis and age group (≤49, 50–59, 60–64, 65–69, ≥70), there was no difference in methylation in semen. BMI did not correlate with CpG1 methylation in seminal plasma. CpG1 methylation in seminal plasma did not differ regarding smoking status and disease.

Positive family history of PCa had 12/30 (40%) PCa patients and 6/27 (22%) BPH patients, but positive family history was not associated with PCa diagnosis (*P* ═ 0.168). Positive family history of any cancer, including PCa had 24/32 (75%) PCa patients and 24/31 (61%) BPH patients but PCa diagnose was independent of positive family history (*P* ═ 0.287).

## Discussion

PCa is the second leading cause of cancer-related death in males but also a silent cancer without early clinical signs of the disease. It is often an indolent disease without need for therapy but the other side of the spectrum is very aggressive behavior needing rapid surgical intervention and oncology care. Due to these facts, screening model for the disease, as well as marker for aggressive forms is needed. Currently, widely used diagnostic and prognostic biomarker PSA show low specificity leading to possible missed diagnosis, and on the other hand, to over-diagnosis of indolent forms of the tumor [19, 20]. Furthermore, PSA cannot discriminate between biologically indolent and aggressive cancer type. Diagnostic biomarker with better performances, reducing unnecessary prostate biopsies, and recognizing clinically significant tumor is subject of scientific community effort [19].

We investigated cfDNA as diagnostic target since it originates from tumor cells and their surrounding cells and can be obtained using minimally invasive techniques. In our study, it is blood and semen. Blood is commonly used in diagnostics but low in cfDNA concentration while semen is, partly, secreted from the prostate and high in cfDNA concentrations [21, 22]. Considering the variety of genetic alternations in PCa (113 mutations in 72 driver gene loci), we focused on epigenetic modifications which are restricted to promoters and retain specific patterns within the same cancer model [23–25]. DNA methylation microarray analysis containing 385,000 probes revealed 20 most significantly altered loci comparing PCa tissue and prostate tissue from BPH patients [26]. One locus was located in *CAV1* gene. Our results showed significantly higher *CAV1* expression in BPH than PCa stroma. Expression in NTT stroma was similar to BPH and also significantly higher than in PCa. This finding was in agreement with known results and confirmed important role of CAV1 in creating changes in PCa microenvironment [27, 28].

Although Di Vizio et al. [28] found that decrease of stromal CAV1 was correlated with higher Gleason score, higher epithelial CAV1 and higher epithelial phospho-Akt. We did not find any correlation between stromal CAV1 and Gleason score, but it is worth saying that our PCa cohort consisted mostly of GGG2 (26/40) and localized disease. Di Vizio et al. [28] cohort focused on metastatic disease. It is possible that high expression of *CAV1* is correlated with stromal changes directing toward more aggressive pro-tumor microenvironment. Absence of epithelial staining in PCa and BPH samples could be due to small number of high grade Gleason tumors, or, as reported by Hamarsten et al., due to high primary antibody dilution since they noticed increased epithelium staining after using less diluted antibody [28]. Furthermore, *CAV1* gene is located at 7q31.1, a region that is deleted in some PCa [29]. Staining score did not correlate with tPSA, which is in concordance with other studies [7].

Compared to adjacent normal tissue, *CAV1* is one of the top ten hypermethylated genes in PCa [30]. *CAV1* methylation in prostate and normal tissue data of Genomic Data Commons TCGA Prostate Cancer (GDC TCGA PRAD) cohort were visualized by the UCSC Xena browser (xenabrowser.net), and the region with the most difference in methylation between groups was selected for our study. In GDC TCGA PRAD study, the mean methylation of each CpGs was higher in PCa tissue. In our study, the most pronounced difference was found for CpG3 which was not analyzed in GDC TCGA PRAD while they showed a statistically significant difference in all seven CpG sites we have in common. In our study, difference was found for two CpGs. Such difference can be explained with the large number of samples (*N* ═ 623) and different methodology used to assess methylation. Furthermore, PCa and BPH tissues have different stroma–epithelial ratios but we did not separate them [31]. The study analyzing *CAV1* methylation 206 kb downstream of our sequence (chr7:116,730,563- 116,730,672) showed that methylation was the highest in PCa tissue, lower in tumor-adjacent tissue, and the lowest in NTT for nine of ten analyzed CpGs [26]. Furthermore, *CAV1* methylation detected cancer in false negative biopsies (AUC 0.70) [32]. Studies analyzing tissue 2 mm and 1 cm away from PCa showed hypermethylation in cancer tissue with less methylated pattern in surrounding tissue, but the distance was irrelevant [31].

**Table 5:** Correlation of tissue methylation with blood and seminal plasma methylation

			**Blood**	**Ejaculate**	**Blood**	**Ejaculate**
			**Sperman *r***		***p***	
CpG1	gDNA (PC+ BPH)		0.02	0.14	0.88	0.27
	gDNA (NTT+ BPH)		0.03	0.07	0.83	0.57
CpG2	gDNA (PC+ BPH)		0.07	0.14	0.55	0.27
	gDNA (NTT+ BPH)		0.10	0.12	0.43	0.36
CpG3	gDNA (PC+ BPH)		−0.08	0.08	0.49	0.53
	gDNA (NTT+ BPH)		−0.02	0.12	0.90	0.36
CpG4	gDNA (PC+ BPH)		−0.05	0.18	0.66	0.15
	gDNA (NTT+ BPH)		−0.08	0.09	0.53	0.49
CpG5	gDNA (PC+ BPH)		0.03	0.15	0.83	0.23
	gDNA (NTT+ BPH)		−0.03	0.20	0.84	0.13
CpG6	gDNA (PC+ BPH)		0.18	0.11	0.14	0.36
	gDNA (NTT+ BPH)		0.04	0.11	0.75	0.42
CpG7	gDNA (PC+ BPH)		0.09	0.29	0.48	0.02
	gDNA (NTT+ BPH)		−0.20	0.31	0.11	0.02
CpG8	gDNA (PC+ BPH)		0.15	0.27	0.22	0.03
	Gdna (NTT+ BPH)		−0.01	0.41	0.95	0.00
CpG9	gDNA (PC+ BPH)		0.19	0.07	0.12	0.60
	gDNA (NTT+ BPH)		0.08	0.05	0.54	0.69
Average	gDNA (PC+ BPH)		0.13	0.09	0.30	0.45
	gDNA (NTT+ BPH)		0.02	−0.02	0.89	0.88

BPH: Benign prostatic hyperplasia; PCa: Prostate cancer; NTT: Non-tumorous tissue.

We did not find any correlation between staining score and tissue methylation, which corresponds to results of Yang et al. [26]. Although many studies have examined methylation of cfDNA in blood and urine as potential biomarkers for PCa, none of them included methylation of *CAV1* [33]. To our knowledge, this is the first study exploring CAV1 methylation in cfDNA from blood and seminal plasma. *CAV1* methylation in blood plasma was not able to distinguish BPH from PCa and did not correlate with gDNA methylation. There are two presumed reasons: cfDNA origin and degradation. The predominant source of cfDNA in the blood of healthy individuals are hematopoietic cells and prostate is an organ were crossing of cfDNA in the blood is low [18, 34]. Chen et al. [5] quantified concentration of cfDNA in blood plasma and reported that patients with metastatic PCa had higher concentrations (median 13.8 ng/ml) than healthy controls (median 7.9 ng/ml), while concentrations of patients with the localized disease were lower than both groups (median 6.7 ng/ml). Our cohort consisted of the localized PCa so it is possible that the amount of cfDNA from prostate in blood plasma is negligible. Secondly, cfDNA elimination from blood is influenced by its form (methylated cfDNA is eliminated slower from the blood than unmethylated) and DNA hydrolyzing activity which is low in blood of PCa patients [34, 35].

Semen is a promising source of PCa biomarker since it is partly produced by prostate. Ponti et al. [36–38] focused their researches on cfDNA from seminal plasma and reported significantly higher cfDNA concentrations in PCa patients, higher concentrations of long fragments (>1000 bp), and different electrophoretic pattern. We explored *CAV1* methylation and found that methylation of CpG1 in seminal plasma was higher in BPH group and superior to serum PSA for distinguishing BPH from PCa (AUC 0.63 vs. AUC 0.52). According to Eggener et al. [3], PCa grade group 1 could be considered indolent PCa so we evaluated competence of seminal *CAV1* methylation to discriminate BPH and PCa GGG1 from PCa GGG ≥ 2. Our results imply that seminal CpG1 methylation may distinguish between BPH and indolent cancer form from PCa with potential aggressive behavior (AUC 0.72) while PSA performance was the same as for distinguishing BPH from PCa (AUC 0.52). By identifying those with aggressive form, one part of men will avoid unnecessary biopsy and overtreatment and *CAV1* methylation could be useful. It is expected that cfDNA originating from prostate is actually from epithelial cells not from stromal. Unfortunately, we did not dissect epithelial cells from stromal cells for methylation analysis, but analyzed it together.

Tissue methylation result (significantly lower methylation in NTT tissue than BPH, no difference with PCa) and gDNA-cfDNA correlation analysis indicate that cfDNA could originate less from PCa and more from NTT cells which released their DNA due to hypoxia or antitumor response. Since seminal plasma in not solely produced by prostate, it is also possible that cfDNA from other tissues confound the result and thus prostate enrichment could be crucial. One study showed that *CAV1* is more expressed in senescent fibroblast while downregulation of *CAV1* expression resulted with cell cycle reentry into S phase. For that reason, we assessed if *CAV1* methylation is associated with age and diagnosis [39]. There was no correlation between age and methylation in seminal plasma. CpG1 methylation in seminal plasma was not changed by age, BMI, or smoking status, which is an important characteristic of good biomarker. Although CpG1 methylation showed better performances than the currently used biomarker, it should be further explored, possible as part of the biomarkers panel. It is also possible to enrich sample with prostate-derived cfDNA.

Corbetta et al. [40] demonstrated an increase in blood cfDNA 1 h (median 3.62 ng/ml) and 2 h (median 7.05 ng/ml) after prostate biopsy was performed. Exercise and inflammation also increase cfDNA concentrations in the blood [40]. Although prostate biopsy is invasive and it is absurdly to use it for increasing cfDNA, prostate massage before ejaculation could be effective. Alternatively, due to risk of false negative biopsy result, *CAV1* cfDNA methylation of CpG1 could be used as a complementary method for tissue biopsies.

Furthermore, many studies identified preanalytical factors that induce variability in blood-derived cfDNA research: fasting status, collection needle gauge, collection tube type, time passed before centrifugation, tube agitation, plasma and cfDNA storage, and quantification [41–43]. Although many studies contributed to realizing cfDNA behavior in blood, to our knowledge, there are no similar studies done on semen. It is to expect that prostate massage would lead to an increase in prostate-derived cfDNA in semen while in-detailed standardized protocol would contribute to reproducibility and ability to compare results from different studies.

## Conclusion

Results from our research indicate that seminal *CAV1* methylation of the analyzed region is able to distinguish BPH and indolent PCa (GGG1) from PCa with potential for aggressive behavior. To use its full PCa biomarker potential, it should be combined with other biomarkers in a panel. Further research expanding general knowledge about cfDNA origin and behavior in semen is needed. A study on a larger cohort with more standardized sampling (prostatic massage, no exercise, fasting condition) could give the wanted result. Due to different stroma–epithelial ratios (BPH 5:1, PCa 4:1), knowledge about tissue methylation in separated compartments and cell types is desirable (stromal and epithelial cells from PCa, surrounding nontumorous tissue, and BPH).

## Acknowledgments

The authors would like to thank Mr. Tomislav Beus for help with microphotography.

**Conflicts of interest:** The authors declare no conflicts of interest.

**Funding:** This research was funded by Croatian Science Foundation, grant number UIP-2017-05-8138 and supported by Scientific Center of Excellence for Reproductive and Regenerative Medicine, the European Union through the European Regional Development Fund, under grant agreement no. KK.01.1.1.01.0008, project “Reproductive and Regenerative Medicine—Exploring New Platforms and Potentials”, and School of Medicine University of Zagreb.

## Figures and Tables

**Table S1:** CpG analyzed in this study and corresponding G Illumina cg designations

**CpG position in this study**	**CG ID**
1	cg07838272
2	cg17469978
3	-
4	cg24980893
5	cg14582226
6	cg23973417
7	cg13522244
8	cg07964538
9	-
